# Pressure injury prevention measures: overview of systematic reviews

**DOI:** 10.1590/1980-220X-REEUSP-2023-0039en

**Published:** 2023-12-22

**Authors:** Franciele Soares Pott, Marineli Joaquim Meier, Janislei Giseli Dorociaki Stocco, Francislene de Fátima Cordeiro Petz, Hellen Roehrs, Patricia Klarmann Ziegelmann

**Affiliations:** 1 Universidade Federal do Paraná, Curitiba, PR, Brazil.; 2 Universidade Federal do Paraná, Complexo do Hospital de Clínicas, Curitiba, PR, Brazil.; 3 Universidade Federal do Rio Grande do Sul, Porto Alegre, RS, Brazil.

**Keywords:** Evidence-Based Nursing, Pressure Injury, Review, Nursing, Wounds and Injuries, Enfermería basada en la evidencia, Úlcera por Presión, Revisión, Enfermería, Heridas y lesiones, Enfermagem Baseada em Evidências, Lesão por Pressão, Revisão, Enfermagem, Ferimentos e Lesões

## Abstract

**Objective:**

Summarizing the evidence from systematic reviews regarding the comparison the effectiveness of interventions to prevent pressure injuries.

**Method:**

Overview of systematic reviews conducted in accordance with *Cochrane* guidelines. A search was performed in databases, repositories and systematic review registration sites.

**Results:**

15 reviews were included in this overview. The sensitivity analysis showed a reduction in the incidence of pressure injuries with nutritional supplementation compared to the standard hospital diet (Relative Risk (RR) = 0.83; 95% Confidence Interval (CI): 0.72–0.95). There was evidence of the superiority of constant low-pressure surfaces (RR = 0.38; 95% CI;0.24–0.61), alternating pressure devices (RR = 0.31; 95% CI:0.17–0.58) and alternative foams (RR = 0.40; 95% CI:0.21–0.74) when compared to the standard hospital mattress or standard foam. The use of a silicone cover reduced the incidence of pressure injuries by 75% (RR = 0.25; 95%CI:0.16–0.41) when compared to no cover.

**Conclusion:**

Although some interventions have been shown to be effective in reducing the incidence of pressure injury, the evidence is limited or very limited and subject to change. Registration CRD42017064586.

## RESUMO

**Objetivo:**

Sumarizar as evidências de revisões sistemáticas sobre a comparação da efetividade de intervenções para prevenção de lesão por pressão.

**Método:**

*Overview* de revisões sistemáticas conduzida de acordo com as recomendações *Cochrane*. Realizou-se busca em bases de dados, repositórios e site de registro de revisões sistemáticas.

**Resultados:**

Foram incluídas 15 revisões nesta *overview*. A análise de sensibilidade demonstrou redução na incidência de lesão por pressão com a suplementação nutricional comparada a dieta hospitalar padrão (Risco Relativo (RR) = 0,83; Intervalo de Confiança(IC) 95%:0,72–0,95). Evidenciaram-se superioridade das superfícies de baixa pressão constante (RR = 0,38; IC 95%;0,24–0,61), dos dispositivos de pressão alternada (RR = 0,31; IC95%:0,17–0,58) e das espumas alternativas (RR = 0,40; IC95%:0,21–0,74) quando comparadas ao colchão hospitalar padrão ou de espuma padrão. O uso de cobertura de silicone reduziu em 75% a incidência de lesão por pressão (RR = 0,25; IC95%:0,16–0,41) quando comparada a nenhuma cobertura.

**Conclusão:**

Embora algumas intervenções demonstrem-se efetivas na redução da incidência da lesão por pressão, as evidências são limitadas ou muito limitadas e sujeitas a alteração. Registro CRD42017064586.

## RESUMEN

**Objetivo:**

Resumir las pruebas de las revisiones sistemáticas sobre la comparación de la efectividad de las intervenciones para prevenir las lesiones por presión.

**Método:**

Resumen de las revisiones sistemáticas realizadas de acuerdo con las recomendaciones Cochrane. Se realizó una búsqueda en bases de datos, repositorios y sitios de registro de revisiones sistemáticas.

**Resultados:**

Se incluyeron 15 revisiones en esta revisión. El análisis de sensibilidad mostró una reducción de la incidencia de lesiones por presión con la suplementación nutricional en comparación con la dieta hospitalaria estándar (riesgo relativo [RR] = 0,83; intervalo de confianza [IC] del 95%: 0,72–0,95). Hubo pruebas de la superioridad de las superficies de baja presión constante (RR=0,38; IC del 95%:0,24–0,61), los dispositivos de presión alternante (RR = 0,31; IC del 95%:0,17–0,58) y las espumas alternativas (RR = 0,40; IC del 95%:0,21–0,74) en comparación con el colchón hospitalario estándar o la espuma estándar. El uso de una funda de silicona redujo la incidencia de lesiones por presión en un 75% (RR = 0,25; IC del 95%:0,16–0,41) en comparación con la ausencia de funda.

**Conclusión:**

Aunque algunas intervenciones han demostrado ser eficaces para reducir la incidencia de lesiones por presión, las pruebas son limitadas o muy limitadas y están sujetas a cambios. Registro CRD42017064586.

## INTRODUCTION

Pressure injuries (PI) are a frequent public health problem worldwide, having prevalence rates varying up to 72.5% in different clinical and geographical contexts. They represent a complication to which many patients are susceptible. The injury is painful, financially costly and negatively impacts the quality of life of the patient and their caregivers, and is mostly preventable^(1)^.

The etiology of PI is multifactorial, involving patient and environmental conditions. A clinical guideline for the prevention and treatment of pressure injuries mentions a study that classifies the relevant risk factors for PI into two groups: mechanical conditions and the person’s susceptibility or tolerance. Mechanical conditions include the magnitude and duration of the mechanical forces applied and their mode of action (compression or using shears). The second group is the individual’s susceptibility and tolerance, which covers internal anatomy (prominence of bone structures, tissue morphology, mechanical and thermal properties of tissues, repair and transport capacity)^(1,2)^.

Given the account of the risk factors, understanding them leads to the adoption of a set of measures (actions) to minimize and/or eliminate the risk factors involved in the occurrence of PI – prevention^(3)^.

In order to help health professionals, especially nurses, to make decisions about preventing PI, international organizations such as *the National Pressure Injury Advisory Panel* (NPIAP), the *European Pressure Ulcer Advisory Panel* (EPUAP), the *Pan Pacific Pressure Injury Alliance* (PPPIA), the *National Institute for Health and Clinical Excellence* (NICE) and the *Wound Ostomy and Continence Nurses Society* (WOCN) put together clinical guides, internationally known as “guidelines”, which are a set of evidence-based recommendations for the treatment and prevention of PI in clinical practice.

In the most recent update of their guideline^(1)^ in 2019, NPIAP, EPUAP and PPPIA indicate three fundamental pillars in the prevention of PI: risk factors and risk assessment; skin and tissue assessment; and preventive skin care. The organizations reinforce that interventions for injury prevention focus on five areas of care: nutrition, repositioning and early mobilization, heel pressure injury, support surfaces and injuries related to medical devices.

In order to identify interventions to prevent PI and their effectiveness in clinical practice, this research aimed to summarize the evidence from systematic reviews on the comparison of interventions to prevent pressure injuries in the general population. In a previous search for overviews of the same nature, no studies were identified that addressed all prevention interventions. One study addresses some support surfaces (beds, mattresses and overlays) in the prevention and treatment of PI^(4)^, which justifies this overview. It should be noted that this publication did not restrict the language or geographical region of the primary research included. It should be noted that the registration of this overview predates the abovementioned publication.

## METHOD

### Type of Study

This is an Overview of Systematic Reviews (SR) conducted in accordance with the recommendations of the *Cochrane Handbook* ^(5)^. The overview aims to compile and synthesize the evidence from multiple systematic reviews and address the effects of more than one intervention on the same health problem^(5)^. The stages were: drafting the research question, defining the inclusion criteria, locating and selecting the SRs, extracting the data, assessing the quality and risk of bias of the SRs included and analyzing and presenting the results.

For the research question, the PICOS strategy was used: Population (P) = children, adults and the elderly at risk of developing PI; Intervention (I) = any intervention or combination of interventions to prevent PI applied in any care setting; Comparison (C) = any other intervention or no intervention; Outcome (O) = incidence of PI and Studies (S) = systematic review of randomized controlled clinical trials, quasi-randomized or cluster-randomized, with no time frame limits.

The following question was defined: What is the evidence from systematic reviews on comparing the effectiveness of interventions to prevent pressure injuries, compared to each other or to no intervention, in the population of children, adults and the elderly, in any care setting?

The protocol for this overview was registered on the *International Prospective Register of Systematic Reviews* (PROSPERO) platform under the number CRD42017064586^(6)^.

### Inclusion Criteria

This overview included Cochrane SRs and non-Cochrane SRs that met the criteria: SR of randomized, quasi-randomized or cluster-randomized controlled clinical trials of any intervention for the prevention of PI, in people of any age and at risk for developing the lesion (assessed using risk assessment scales and/or clinical evaluation). For the non-Cochrane SRs on PI prevention, we considered the use of a systematic method, with a comprehensive and detailed search strategy; clear definition of the selection criteria for the primary studies; evaluation of the methodological aspects of the studies included and reporting and synthesis of the evidence identified.

Studies that discussed, in addition to prevention results, data related to the treatment of PI, were included only if the prevention results, the object of interest, were presented separately. There were no restrictions on language, year of publication or place of care.

We excluded SRs that used a definition of PI that was not based on validated sources and those that included the term systematic review in the title, but did not follow the rigor of the method.

### Location and Selection

For all the databases consulted, an electronic search was carried out in July/2017 with updates in January/2018, November/2019, October/2020, August/2021 and May/2023, in five databases: *Medical Literature Analysis and Retrieval System Online* (MEDLINE/PUBMED); *Excerpta Medica Database* (EMBASE); *Cochrane Database of Systematic Reviews* (CDSR); *Database of Abstracts of Reviews of Effects Cochrane* (DARE Cochrane); *Health Technology Assessment Database*. The search strategies used the official terms and their synonyms from the *Medical Subject Headings* (MESH) and *Embase Subject Headings* (EMTREE), as well as words that identified the interventions studied. Repositories and websites of SR registries were also consulted on the PROSPERO platform. The search strategy adopted for the MEDLINE database, which was adapted for the other databases analyzed is shown in Chart 1.

**Chart 1:** MEDLINE database search strategy – Curitiba, PR, Brazil, 2023.

((((((((((((“pressure ulcer”[MeSH Terms])) OR “pressure sore*”[Text Word]) OR “decubitus ulcer*”[Text Word]) OR “decubitus sore*”[Text Word]) OR “bed sore*”[Text Word]))) OR ((“pressure ulcer/prevention and control”[MeSH Terms])))) AND ((((((((((“skin care”[MeSH Terms]) OR “skin care/methods”[MeSH Terms]) OR “skin evaluation”[Text Word]) OR “skin assessment”[Text Word]) OR “risk assessment”[MeSH Terms]) OR “risk assessment/methods”[MeSH Terms])) OR ((((“enteral nutrition”[MeSH Terms]) OR “enteral nutrition”[Text Word]) OR “parenteral nutrition”[MeSH Terms]) OR “parenteral nutrition”[Text Word])) OR (((((((((((“reposition*”[Text Word]) OR “re position”[Text Word]) OR “position”[Text Word]) OR “turn patients”[Text Word]) OR “turn intervals”[Text Word]) OR “turn frequen*”[Text Word]) OR “body postur*”[Text Word]) OR “turning”[Text Word]) OR “mobilis*”[Text Word]) OR “mobiliz*”[Text Word]) OR (“moving and lifting patients”[Text Word]))) OR (((((“pressure relief”[Text Word]) OR “pressure relieve”[Text Word]) OR “pressure reliev*”[Text Word]) OR “pressure reduction”[Text Word]) OR “pressure alleviation”[Text Word])))) AND ((((((((((((((((((((((“meta analysis as topic”[MeSH Terms]) OR “meta analysis”[Text Word]) OR “meta analysis”[Publication Type]) OR “review literature as topic”[MeSH Terms]) OR “review literatures”[Text Word]) OR “review”[Publication Type]) OR “review”[Text Word]) OR “systematic* review*”[Text Word]) OR “synthes* literature”[Text Word]) OR “synthes* evidence”[Text Word]) OR “integrative review”[Text Word]) OR “data synthesis”[Text Word]) OR “research synthesis”[Text Word]) OR “narrative synthesis”[Text Word]) OR “systematic study”[Text Word]) OR “systematic studies”[Text Word]) OR “systematic comparison”[Text Word]) OR “evidence based review”[Text Word]) OR “meta-analytic*”[Text Word]) OR “meta-analysis”[Text Word]) OR “metanalysis”[Text Word]) OR “metaanalysis”[Text Word])

The SRs were selected independently by two reviewers (FSP and JS) based on the inclusion/exclusion criteria previously established. The same pair of reviewers took part in the reading of the titles and abstracts and the reading of the full texts. At both selection stages, disagreements were discussed by a third reviewer (MM).

### Data Collection

For the purposes of data extraction, a pre-defined instrument created by the authors was used, which included data on the identification of the review, last update, authors, objectives of the review, care setting, inclusion and exclusion criteria, population included, number of Randomized Clinical Trials (RCTs) included, comparisons, results reported and how the risk of bias/methodological quality was assessed.

### Assessment of Methodological Quality

The methodological rigor of the SRs included in this study was assessed using the AMSTAR 2^(7)^ tool (*Assessment of Multiple Systematic Reviews*). With regard to the quality of the evidence, the results were presented using the GRADE assessment (*Grades of Recommendation, Assessment, Development and Evaluation*) when this analysis was described by the SR. In the case of extra analyses carried out by the authors of this overview, GRADE was prepared on a case-by-case basis using the GRADEpro GDT (*Profiler, Guideline Development Tool)* software, according to its classification (lowest, low, moderate and high)

### Data Analysis and Processing

The PI prevention interventions evaluated in the reviews included in this overview were classified according to the categories proposed by the NPIAP, EPUAP and PPPIA guidelines^(1)^.

To summarize the data, the results were described as presented by each SR^(8)^. In specific cases, sensitivity analyses were conducted by the authors of this overview, with the following criteria: in the nutritional support intervention, primary studies that had a population of more than 80% malnourished and/or risk of bias in more than one domain were excluded.

## RESULTS

**Figure 1:**
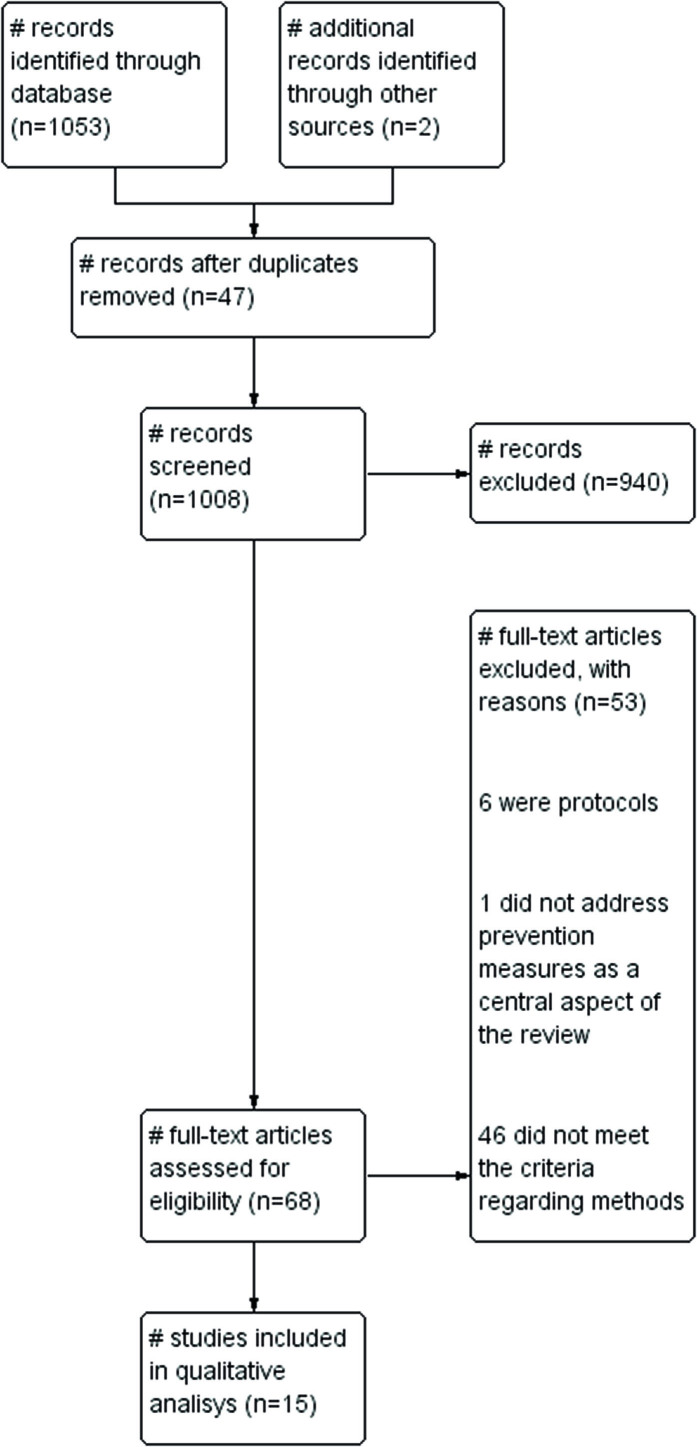
Flowchart for the identification, selection and inclusion of Systematic Reviews according to the Prisma criteria – Curitiba, PR, Brazil 2023.

A total of 1053 titles were identified through the database searches, as well as two additional records of uncompleted protocols. After reading the titles and abstracts, 68 SRs were selected for full reading, of which 15 met the eligibility criteria and were included in this overview (Figure 1)

The 15 reviews^(9–23)^ analyzed in this overview involved a total of 61,527 participants. The publications took place between 2006 and 2022, with one publication in 2006, one in 2014, two in 2015, 2018, 2019, 2020 and 2022 and four in 2021. However, the publication dates of the primary studies in the SRs are variable, as there was no time frame in some of these reviews. In 13 SRs^(9–15,17–22)^, the investigated preventive measures fell into one of the categories proposed in the analysis: risk assessment; nutritional assessment and support, use of support surfaces, repositioning and mobilization and other interventions to prevent PI (protective coverings, massage, specialized staff and exercise and incontinence care). The SR8^(16)^ included preventive measures in several categories. It is noteworthy that there are three reviews SR6^(14)^, SR7^(15)^ and SR14^(22)^ that did not have studies included based on the selection criteria.

In the SR8^(16)^ review, there was an overlap of primary studies, with their results described by more than one included review, so only the “exercise and incontinence care” intervention was analyzed in this overview. Reviews SR10^(18)^, SR11^(19)^, SR12^(20)^, SR13^(21)^ and SR14^(22)^ also found overlapping primary studies, so for comparisons with overlapping studies, the results were presented only once.

To assess the risk of bias, 13 SRs^(9–15,18–23)^ used the Cochrane Risk of Bias Tool, one^(16)^ used a specific checklist, which assessed the quality of reports of RCTs of non-pharmacological interventions based on six elements: adequate generation of allocation sequence; concealment of treatment allocation; adequate blinding of the participant; adequate blinding of the evaluator; consistent follow-up schedule and intention-to-treat analysis. SR9^(17)^ only reports the use of the Jadad scale to assess the methodological quality of the included studies.

Regarding methodological quality, according to *AMSTAR*2, seven reviews^(11,13,18–22)^ were classified as high, five^(9,10,12,14,15)^ as moderate and three^(16,17,23)^ as low quality. All^(9–23)^ evaluated the outcome of PI incidence.

The analysis of the comparisons studied is displayed in Table 1. Table 2 is for SR3^(11)^, which has many comparisons, and SR10^(18)^, SR11^(19)^, SR12^(20)^, SR13^(21)^, SR14^(22)^ and SR15^(23)^, which have interventions in the same category. Most of the analyses showed no statistically significant difference in the incidence of PI, and those that were significant will be discussed further below.

**Table 1:** Results of comparisons between pressure injury prevention measures as reported by the original SRs – Curitiba, PR, Brazil 2021.

Identification of SR/Category/No. of studies included/Total no. of participants	Comparison of Interventions	n	RR (CI 95%)
SR1^(9)^	Braden Scale × Training + Unstructured	150	0.97 (0.53 – 1.77)
“Risk Assessment	Braden Scale × Unstructured	180	1.43 (0.77 – 2.68)
# 2	Waterlow Scale × Unstructured	821	1.10 (0.68 – 1.81)
1487	Ramstadius Scale × Unstructured	820	0.79 (0.46 – 1.35)
	Waterlow Scale × Ramstadius Scale	831	1.44 (0.85 – 2.44)
SR2^(10)^	Mixed Nutritional Supplement × Standard Hospital Diet	6064	0.86 (0.73 – 1.00)
“Nutritional Assessment and Support”	Mixed Nutritional Supplement (FE TAG) × DEP (ATC)	30	0.77 (0.37 – 1.57)
#11	Mixed Nutritional Supplement (FE ATG BTC EL) × SNM (FE ATG BTC)	95	0.85 (0.37 – 1.97)
6605			
SR4^(12)^	Repositioning: 2h × 4h (PI grade 1 to 4); any support surface)	1074	1.06 (0.80 – 1.41)
“Repositioning and Mobilization”	Repositioning: 2h × 3h (pressure injury grade 1 to 4); HP mattress	129	0.90 (0.69 – 1.16)
# 8	Repositioning: 2h × 3h (grade 2 to 4 pressure injuries);	252	0.59 (0.28 – 1.26)
3941	Repositioning: 2h × 3h ; High density foam	967	4.06 (0.87 – 18.98)
	Repositioning: 3h × 4h; High density foam	407	0.20 (0.04 – 0.92)
	Repositioning: 4h × 6h Viscoelastic mattress (PI grade 1 to 4)	129	0.73 (0.53 – 1.02)
	Repositioning: 30° 3h × 90° (overnight)	252	0.62 (0.10 – 3.97)
	Repositioning: 2h 20º × “standard treatment”	1312	0.28 (0.10 a 0.75)
	Head-of-bed tilt at 30º × 45º (Mobilization every 2h)	120	–
	Prone position × supine position	116	–
SR5^(13)^	Fatty Acid × Olive Oil	1060	1.28 (0.76 – 2.17)
“Protective Covers (Other Interventions)”	Fatty acid × Control compound	331	0.42 (0.22 – 0.80)
#18	Fatty acid × Standard treatment	187	0.70 (0.41 – 1.18)
3629	Active lotion × placebo/control	1,67	0.73 (0.45 – 1.19)
	DMSO-Cream × placebo/control	61	1.99 (1.10 – 3.57)
	Conotrane × placebo/control	258	0.74 (0.52 – 1.07)
	Mepentol × placebo/control	331	0.42 (0.22 – 0.80)
	Prevasore × placebo/control	120	0.33 (0.04 – 3.11)
	Silicone cover vs. no cover	1246	0.25 (0.16 – 0.41)
	Polyurethane film vs. hydrocolloid	160	0.58 (0.24 – 1.41)
	Kang’ huier × care routine	100	0.42 (0.08 – 2.05)
	PPD × no coverage	74	0.18 (004 – 0.76)
	Thin polyurethane foam × no coverage	74	1.31 (0.83 – 2.07]
	Adhesive foam cover × no cover	78	1.65 (1.10 – 2.48)
SR6^(14)^ “Massage (Other interventions)” # 0
	**Empty review**	**0**	**–**
	No randomized or quasi-randomized clinical trials comparing massage with placebo, standard treatment or other therapies were identified by the review authors		
			
SR7^(15)^ “Specialized team (Other interventions)” **# 0**
	**Empty review**	**0**	**–**
	No studies were included in the review because they did not meet the inclusion criteria pre-established by the authors		
		
SR8^(16)^	Exercises + Incontinence Care vs. Usual Care	144	0.88 (0.41 – 1.91)
“Exercises + Incontinence Care (Other Interventions)”			
# 59			
144			
SR9^(17)^	Hydrocolloid cover vs. control	2519	0.22 (0.17 – 0.29)
“Protective Covers (Other Interventions)”	Hydrocolloid cover × Control (Children)	626	0.11 (0.04 – 0.29)
# 22	Hydrocolloid cover × Control (Adults and elderly)	1893	0.24 (0.19 – 0.31)
2519	Hydrocolloid cover × Gauze	908	0.26 (0.17 – 0.38)
	Hydrocolloid cover × Skin care	1611	0.21 (0.15 – 0.29)

SR – Systematic review; n – sample number; RR – Relative risk; FE TAG – Abnormal glucose tolerance enteral formula; DEP – Standard enteral diet; ATC – High carbohydrate content; FE ATG BTC EL – High-fat, low-carbohydrate enteral formula enriched with lipids; SNM – Mixed nutritional supplement; FE ATG BTC – High-fat, low-carbohydrate enteral formula; HP – Hospital Standard; Dermalex™ – active lotion containing: hexachlorophane 0.5%, saturated hydrocarbons (squalene (Cosbiol 3%) and glyoxyl diureide), allantoin 0.2%, antioxidants, lanolin, fatty acids, fatty acid esters, fatty alcohols, preservatives and distilled water; DMSO – Cream – consisting of 5% dimethylsulfoxide in vaseline – ketomacrogol cream; Conotrane – silicone cream; 20% dimethicone 350; and a broad – spectrum antiseptic (0.05% hydrargafen); Mepentol – hyperoxygenated fatty acid compound (consisting of: oleic acid, palmitic acid, stearic acid, palmitoleic acid, linoleic acid, gamma – linoleic acid, arachidonic acid and eicosenoic acid; Prevasore – Prevasore (Hexyl nicotinate, zinc stearate, isopropyl myristate, dimethicone 350, cetrimide and glycol); CE – External layer; FN – Nylon fibers. PPD – PPD dressing (pressure ulcer prevention dressing) with an adhesive layer for the skin (hydrocolloid), a support layer (urethane film) and an outer layer of multifilament nylon fibers). Kang ‘huier transparent strip and foam dressing. Conotrane – silicone cream; 20% dimethicone 350; and a broad – spectrum antiseptic (0.05% hydragaphene); Mepentol – hyperoxygenated fatty acid compound (consisting of: oleic acid, palmitic acid, stearic acid, palmitoleic acid, linoleic acid, gamma – linoleic acid, arachidonic acid and eicosenoic acid; Prevasore – Hexyl nicotinate, zinc stearate, isopropyl myristate, dimethicone 350, cetrimide and glycol).

**Table 2:** Results of comparisons between pressure injury prevention measures as reported by Review 3 – Curitiba, PR, Brazil 2021.

Identification of SR/Category/No. of studies included/Total no. of participants	Comparison	Studies	n	RR
SR3^(11)^	Constant Low Pressure x Standard Hospital Mattress	7	2407	0.38 (0.24 – 0.61)
“Support Surfaces”	Alternative Foam x Standard Foam Mattress	5	2016	0.40 (0.21 – 0.74)
# 59	Alternative Foam x Standard Foam	1	505	0.36 (0.22 – 0.59)
12624	Foam mattress (MAXIFLOAT) x Foam overlay	11	40	0.42 (0.18 – 0.96)
	Solid foam x convoluted foam		84	0.66 (0.37 – 1.16)
	Transfoam mattress x Transfoam wave mattress	1	100	1.00 (0.06 – 15.55)
	Cold foam mattress x cold foam mattress and static air overlay	1	83	3.59 (0.79 – 16.25)
	Constant Low-Pressure Devices	11	2138	0.45 (0.36 – 0.56)
	Optima x Standard foam mattress	1	40	0.06 (0.00 – 0.99)
	Sofflex x ROHO	1	84	0.63 (0.16 – 2.47)
	Gel mattress x Air-filled overlay	1	66	0.80 (0.24 – 2.72)
	Static air x water mattress	1	37	0.43 (0.04 – 4.29)
	Foam overlay x Silicone overlay	1	68	1.17 (0.64 – 2.14)
	Sheepskin x No sheepskin	1	539	0.57 (0.34 – 0.94)
	Sheepskin x No sheepskin	1	297	0.30 (0.17 – 0.52)
	Sheepskin x No sheepskin	1	588	0.60 (0.37 – 0.96)
	Foam support x no support	1	70	0.16 (0.05 – 0.49)
	Heel-lift suspension boot and various SS x SS only	1	239	0.26 (0.12 – 0.53)
	Static inflated vs. Microfluidized static or BPA mattress	1	110	0.33 (0.07 – 1.58)
	Sheepskin vs. no sheepskin (PI grade 2)	3	1424	0.59 (0.33 – 1.05)
	Alternating Pressure x Standard Foam Mattress	2	409	0.31 (0.17 – 0.58)
	Low Pressure Alternating Bed x Standard Bed	2	221	0.33 (0.16 – 0.67)
	Viscoelastic polymer pillow x SS	1	416	0.53 (0.33 – 0.85)
	Micropulse system for surgical patients x standard care	1	368	0.21 (0.06 – 0.70)
SR10^(18)^	Alternating Pressure Air Surface (Active) x Reactive Foam Surface	4	2247	0.63 (0.34 – 1.17)
“Support Surfaces	Alternating Pressure Air Surface (Active) x Reactive Air Surface	6	1648	1.61 (0.90 – 2.88)
# 32	Alternating Pressure Air Surface (Active) x Reactive Water Surface	2	358	1.21 (0.52 – 2.83)
9058	Alternating Pressure Air Surface (Active) x Reactive Fiber Surface	3	285	0.90 (0.68 – 1.19)
	Alternating Pressure Air Surfaces (Active) on operating tables and later on the ward bed x Reactive Gel Surfaces on operating tables and followed by Foam Surfaces on the ward bed	2	415	0.22 (0.06 – 0.76)
SR11^(19)^	Water Reactive Surface vs. Air Reactive Surface	1	37	2.35 (0.23 – 23.75)
“Support Surfaces	Reactive Fiber Surface x Foam Surface	1	68	0.86 (0.47 – 1.57)
# 20	Reactive Gel Surface x Reactive Air Surface	1	66	0.80 (0.36 – 1.77)
4653	Water Reactive Surface x “Undefined” Standard Hospital Surfaces	1	316	0.35 (0.15 – 0.79)
	Reactive Gel Surface x “undefined” Standard Hospital Surfaces	2	446	0.53 (0.33 – 0.85)
SR12^(20^) “Support Surfaces # 17 2604 SR13(21) “Support Surfaces # 29 9566
	Reactive Air Surface x Foam Surface	4	229	0.42 (0.18 – 0.96)
	Reactive Air Surface (KinAir) x Reactive Air Surface (EHOB Waffle) – Two types of Reactive Air Surface	1	123	0.66 (0.29 – 1.49)
	Foam Surface vs. Reactive Gel Surface	1	270	–
	Foam Surface x Reactive Foam Surface and Gel Surface	1	182	–
			
			
			
				
SR14^(22)^ “Support Surfaces” # 0 0
	Empty review	0	–	–
	No studies were included in the review because they did not meet the inclusion criteria pre-established by the authors			
			
				
SR15^(23)^ “Support Surfaces # 6 4697
	Alternating pressure air mattress with repositioning interval every 2 hours x Viscoelastic foam mattress with repositioning interval every 4 hours	1	1194	9.97 (1.28 – 77.61)
	Alternating pressure air mattress vs. static air mattress	1	308	8.22 (0.95 – 4.78)
	Alternating pressure air mattress x Static air mattress	1	1074	0.12 (0.09 – 0.15)
	Alternating pressure air mattress x Static air mattress	1	16	0.15 (0.04 – 0.60)
	Alternating pressure air mattress x High specification foam mattress	1	2029	0.91 (0.28 – 2.98)
	Alternating pressure air mattress x Memory foam mattress	1	76	1.00 (0.18 – 5.46)

SR – Systematic review; n – sample number; RR – Relative risk; SS – Support surface; BPA – Low air loss; PI – Pressure Injury.

In the “*Nutritional assessment and support*” category, a primary study (RCT) carried out only with malnourished patients and with a high risk of bias in the allocation and blinding domains^(24)^ was excluded in the sensitivity analysis of SR2^(10)^, resulting in meta-analysis with seven RCTs and 5525 participants (RR = 0.83, 95%CI:0.72–0.95), which showed a lower incidence of PI in the intervention group. The GRADE analysis considered the level of evidence to be moderate (downgraded because there was a high risk of bias in the following areas: generation of the randomization sequence, allocation or blinding). It should be noted that although the sensitivity analysis reduced the confidence interval, it did not change the direction of the effect estimate.

In the “*Use of Support Surfaces*” category, SR3^(11)^ evaluated different technologies in this intervention group and included 59 studies with a total of 12,624 participants. Support surfaces were classified into three groups: low-tech (which includes constant low-pressure devices such as: sheepskin; static air-filled supports; water-filled support; contoured or textured foam support; gel-filled support; granule-filled support; fiber-filled supports; alternative foam mattresses or overlays), high-tech (PA: alternating pressure supports, low air loss beds and air fluidized beds) and other support surfaces (“kinetic turning table”, “profiling beds”, operating table overlays and seat cushions).

In this same category, “*Use of Support Surfaces*”, SR10^(18)^, SR11^(19)^, SR12^(20)^, SR13^(21)^, SR14^(22)^ and SR15^(23)^ analyzed the effects of different materials on the prevention of PI. The studies in question involved a total of 30,578 participants. Different groups of interventions were compared in terms of the outcome “incidence of PI” and the significant comparisons are displayed in Table 2.

## DISCUSSION

The different strategies for preventing PI have been recommended by international guidelines since the 1990s, with the aim of reducing its incidence (WOCN; NPIAP, EPUAP, PPPIA; NICE)^(1)^.

In order to contribute to nurses’ decision-making and to help incorporate best practices into care, this overview summarized the evidence from 15 available SRs on different pressure injury prevention measures, which investigated the main outcome – PI incidence.

For “*risk assessment*” interventions, studies highlight the sensitivity of scales in predicting PI risk^(25)^. However, no evidence has been found that their use reduces the occurrence of PI^(9)^. The limited number of studies included in the SR1 review^(9)^ and the low methodological quality translate into uncertain conclusions, so that new RCTs may alter the estimated effect of this intervention. Although not proven to be effective in reducing the occurrence of injuries, risk assessment tools are predictors of PI, prompting the early adoption of other prevention strategies^(26)^.

With regard to “Nutritional assessment and support”, the cluster analysis of the studies in SR2^(10)^ showed no statistically significant differences in the occurrence of PI when comparing supplements with the standard hospital diet. There was significant heterogeneity between the interventions in the supplement group due to the different presentations and levels of proteins, vitamins, fats and carbohydrates.

In SR2^(10)^ the studies presented uncertain risk or high risk of bias for important domains, which compromises their quality and, consequently, the certainty of the effect estimates. Thus, the evidence found was considered to be of “very low” quality according to the *GRADE* analysis (very low, low, moderate, high), which suggests that there is a high degree of uncertainty in the findings.

The sensitivity analysis conducted in this overview of the findings of SR2^(10)^ showed that nutritional supplementation can help reduce the incidence of PI (RR = 0.83; 95% CI:0.72–0.95), with a “moderate” degree of certainty in the estimated effect, according to the GRADE assessment. However, further studies could still have an impact on the estimated effect for this intervention, changing the confidence in the estimate or even changing the estimate itself.

Nutrition plays a vital role in the prevention and treatment of pressure injuries, as all organ systems require macro- and micronutrients to meet nutrient needs for growth, development, maintenance and repair of body tissues. According to the latest update of the NPIAP Guideline, the EPUAP and PPPIA^(1)^, well-nourished individuals have a lower risk of developing PI when compared to malnourished individuals. However, it is known that both well-nourished and undernourished individuals can develop skin integrity problems in certain circumstances.

SR4^(12)^, which deals with “*repositioning and mobilization*” interventions, did not provide sufficient evidence to choose which frequency (2h, 3h and 4h) or positions (20º, 30º, 45º, 90º, prone and supine) are most effective in reducing pressure damage. Repositioning every 3 hours versus every 4 hours on a high-density foam mattress was more effective in reducing the incidence of PI (RR = 0.20; 95% CI:0.04–0.92). However, the certainty of the evidence was considered “low” due to the risk of bias and imprecision of the results^(12)^.

Repositioning every 4 hours versus 6 hours on a viscoelastic mattress resulted in a reported 27% reduction in the occurrence of PI (RR = 0.73, 95% CI 0.53–1.02). However, the certainty of the evidence is “very low” due to the high risk of bias in the primary studies included in the systematic review. Limitations were observed in the design (lack of blinding of outcome evaluators and personnel and missing outcome data) and imprecision of the results presented^(12)^.

However, the lack of evidence for repositioning with regard to frequency and specific positions should not be interpreted as evidence of ineffectiveness^(12)^. When considering the etiology of the development of PI, linked to localized vascular obstruction, which reduces capillary blood flow to the skin surface area, there are reasonable grounds to expect that repositioning will minimize the risk of deprivation of oxygen and nutrients that are necessary for maintaining tissue integrity^(10)^.

Of the five SRs^(13–17)^ included in the “Other interventions” category for the prevention of pressure injuries, which evaluated the effectiveness of various preventive measures, two reviews^(13,17)^ analyzed the effects of coverings and/or topical agents in reducing the incidence of PI.

In SR5^(13)^, when comparing different topical agents, the heterogeneity of the interventions did not allow for a pooled analysis. The results presented showed that the incidence of PI was lower with treatment containing fatty acid compared to a control compound (RR 0.42, 95% CI 0.22–0.80), but the evidence was considered to be of “low” certainty due to the serious risk of bias and imprecision^(13)^.

In addition, in SR5^(13)^ one of the topical agents analyzed (DMSO-cream) may increase the risk of PI (n = 61; RR = 1.99; 95% CI 1.10–3.57) compared to placebo; however, the findings were based on a single low-quality study, which reflects the low quality of the evidence. Low quality evidence implies limited confidence in the estimate of effect, i.e. the true effect is likely to be substantially different from the estimate of effect^(12)^.

Additionally, in SR5^(13)^, when comparing silicone coverage versus no coverage, the experimental intervention was significantly superior to the control (RR = 0.25; 95% CI:0.16–0.41). The evidence generated by the study’s meta-analysis was of low quality, so future studies will probably have an important impact on the confidence of the effect estimate^(13)^.

The analysis of the effects of protective coverings on the prevention of PI related to medical devices in SR9^(17)^ showed that hydrocolloid was superior to all the comparators studied and in different age groups (RR = 0.22; 95% CI:0.17–0.29). However, the systematic review does not provide enough information to analyze the quality of the evidence generated.

With regard to the use of these technologies in the prevention of PI, the NPIAP, EPUAP and PPPIA guidelines^(1)^ state that the choice of coverage should consider the following characteristics: the benefit of its use; the appropriateness of its size and design; its ability to manage the microclimate; ease of application and removal; ability to remain fixed at the applied site; ease of handling for skin assessment; being compatible with the patient’s preferences; being comfortable; hypoallergenic; that minimizes the coefficient of friction between the skin-cover interface and the cost effectiveness of the technology^(1)^.

Two reviews (SR6 and SR7)^(14,15)^ in the category “Other interventions” in pressure injury prevention did not include any studies. One looked at the effectiveness of massage^(14)^ and the other at the role of a specialized team^(15)^ in preventing and treating the injury, respectively. As they were considered “empty” reviews, they do not allow conclusions to be drawn about the effectiveness of the interventions analyzed.

SR8^(16)^ in the “Other interventions” category found no significant evidence that the combination of exercise and incontinence care, compared to usual care, reduces the incidence of PI (RR = 0.88; 95%CI:0.41–1.91). It should be noted, however, that the data was based on only one study, of low methodological quality.

The literature shows a moderate statistical association between excessive skin humidity and the occurrence of PI. In addition to exposure to moisture, incontinence culminates in exposure to chemical irritants from feces and urine and consequent inflammation, erythema, erosion and denudation of the tissue, which reduces its tolerance to pressure and use of shears^(1)^.

Seven reviews^(11,18–23)^ were classified in the “*Use of Support Surface*” category. In SR3^(11)^, various technologies were evaluated for their effectiveness in reducing the incidence of PI. The meta-analytical groupings showed the superiority of different devices when compared to the standard, such as low constant pressure support surfaces (RR = 0.38; 95%CI:0.24–0.61), alternating pressure devices (RR = 0.31; 95%CI:0.17–0.58) and alternative foams, known as high specification foams (RR = 0.40; 95%CI:0.21–0.74). However, for the latter, a study that analyzed the certainty of the evidence produced indicated that this result is highly uncertain^(27)^.

SR10^(18)^ showed that Alternating Pressure Air Surfaces (Active), when used on overlapping surgical tables and later in hospital beds, compared to gel overlays on surgical tables and foam overlays in hospital beds, can reduce the incidence of PI (RR = 0.22; 95%CI: 0.06–0.76). However, in the GRADE analysis, the authors considered the evidence to be of low certainty. In the comparison with foams, the authors found that Alternating Pressure Air Surfaces (Active) can reduce the proportion of people who develop PI. For the other comparisons presented, it is uncertain whether there is any difference between alternating pressure (active) air surfaces and the technologies used in the comparison.

In contrast to the findings of this study, a meta-analysis by Network^(27)^ showed that there is moderate certainty that active (motorized) air surfaces and hybrid (motorized) air surfaces can reduce the incidence of PI when compared to the standard hospital mattress (RR = 0.42; 95%CI:0.29–0.63 and RR = 0.22; 95%CI:0.07–0.66), which justifies their adoption in clinical practice. However, new studies may alter the effect estimates.

In SR11^(19)^ the authors compared different alternative reactive support surfaces (without foam and without air) in the prevention of PI and showed that it is still uncertain whether there is a difference in the incidence of the lesion with the technologies studied. In addition, the GRADE analysis showed that the evidence is of very low certainty, so the real effect could be substantially different from the estimated effect.

The SR12^(20)^ showed that the reactive air surface was superior in preventing PI when compared to the foam surface (RR = 0.42; 95%CI: 0.18–0.96), however the GRADE assessment indicated that this result is uncertain and confidence in the estimated effect is limited. In SR13^(21)^, the authors evaluated foams in the prevention of PI, but no statistical analysis was carried out for some of the comparisons presented. However, the authors mention that there was no development of PI in any of the groups studied.

SR14^(22)^ aimed to assess the effects of pressure redistribution in static chairs on the prevention of PI. However, as it is an “empty” review, which did not include any RCTs, it does not allow conclusions to be drawn about the effectiveness of the proposed interventions.

The analysis of the effects of alternating pressure air mattresses compared to static air mattresses in the SR15^(23)^ showed that alternating pressure was superior to control in two comparisons (RR = 0.12; 95% CI:0.09–0.15 and RR = 0.15; 95% CI:0.09–0.60). However, the systematic review does not provide enough information to analyze the quality of the evidence generated.

A study on different support surfaces concurs with the results of this overview by stating that the evidence is unclear regarding the relative effectiveness of most of the available comparisons in relation to the prevention of PI^(4)^. In addition, there is no clarity as to which support surface is the most effective in preventing lesions, as all the evidence found is of very low certainty^(4)^. According to the same authors, there is low-certainty evidence that, compared to foam surfaces (reference technology), reactive air surfaces (static air overlays) (RR = 0.46; 95% CI: 0.29–0.75), alternating pressure (active) air surfaces in general (e.g. alternating pressure air mattresses, large cell corrugated mattresses) (RR = 0.63;95% CI:0.42–0.93) and reactive gel surfaces (e.g. gel pads used on operating tables) (RR = 0.47;95% CI:0.22–1.01) can reduce the incidence of pressure injury^(4)^.

Even with the superiority of some interventions in reducing the incidence of PI, the lack of clear description of the interventions in the primary studies, small sample sizes and different follow-up times contribute to clinical and statistical heterogeneity, and need to be taken into account when interpreting the results^(11)^.

Another point to be considered is that some authors^(9,10,13,15–17,23)^ have not summarized the certainty of the evidence using GRADE, but their considerations make it clear that there are gaps to be elucidated. Thus, new studies could substantially alter the conclusions and certainty of the effect estimates.

It should be noted that there is a risk of bias in primary studies (RCTs) such as: lack of allocation concealment, lack of baseline comparability, high attrition rates, lack of intention-to-treat analysis, and non-blinding of outcome assessment, which compromise the quality of the findings, which in short, does not favor obtaining evidence with a moderate or high level of certainty.

The evidence on the effectiveness of PI prevention measures is still uncertain and may change with the publication of new studies. Therefore, it is essential that future studies adopt standard recommendations for reporting RCTs, as well as systematic reviews (such as PRISMA/2020^(28)^) to ensure the methodological quality of publications, so that new SRs and overviews can reduce these uncertainties and contribute to a clinical practice based on cost-effective evidence.

## CONCLUSION

The results of this overview showed that, although some PI prevention interventions have been shown to be effective in reducing the incidence of lesions, the evidence is still limited or very limited, as it was judged to be of “low or very low” quality. This means that new studies could substantially alter the confidence in the estimate of effect, as there is a significant degree of uncertainty in the findings.

However, nurses (members of the multidisciplinary team and teams specializing in skin care) are advised that “low or very low” quality evidence does not mean ineffectiveness. Professionals should consider the benefits of incorporating it into clinical practice and keep up to date with new evidence and/or the publication of updated guidelines. It should be noted that most of the various PI prevention measures discussed in this overview are recommended by international organizations.

With regard to research reports, it is recommended that they be conducted in accordance with guidelines such as PRISMA and AMSTAR, in order to guarantee their quality. It is suggested that GRADE be used to analyze the results of systematic reviews in order to identify the quality of the evidence produced

It should be noted that it is essential to adopt recommendations such as the Consolidated Standards of Reporting Trials (CONSORT) for conducting RCTs, in order to produce better quality evidence and standardize research reports to ensure that no relevant information is omitted. The low methodological quality of the studies included in SRs has a direct impact on the findings of the reviews, limiting their conclusions and making it impossible to obtain “moderate or high” evidence.
